# End-organ damage from neonatal invasive fungal infection: a 14-year retrospective study from a tertiary center in China

**DOI:** 10.1186/s12879-024-09360-7

**Published:** 2024-05-23

**Authors:** Tao Han, Meng Qiu, Xinxin Niu, Shumei wang, Feng Wang, Jingke Cao, Shanghong Tang, Liping Cheng, Yabo Mei, Huayu Liang, Zhichun Feng, Geyu Chen, Qiuping Li

**Affiliations:** 1 Department of Neonatology, Senior Department of Pediatrics, The Seventh Medical Center of Chinese PLA General Hospital, NO.5 Nanmencang, Dongcheng District, Beijing, 100007 China; 2 National Engineering Laboratory for Birth defects prevention and control of key technology, Beijing, China; 3 Beijing Key Laboratory of Pediatric Organ Failure, Beijing, China; 4 Department of Organ Transplantation, the Third Medical Center of Chinese PLA General Hospital, Beijing, China; 5 The Second School of Clinical Medicine, Southern Medical University, Guangzhou, China; 6 Department of clinical medicine, Nan Fang Hospital affiliated to Southern Medical University, 1838 North Guangzhou Avenue, Baiyun District, Guangzhou, 510515 China

**Keywords:** End-Organ Damage, Invasive fungal infection, Neonates, Fungi, Candida albicans

## Abstract

**Background:**

Invasive fungal infection (IFI) has become an increasing problem in NICU neonates, and end-organ damage (EOD) from IFI is one of the leading causes of morbidity and mortality in neonates. This study was conducted to summarize clinical data on epidemiology, risk factors, causative pathogens, and clinical outcomes of IFI-associated EOD among neonates in a center in China for the sake of providing references for prevention and treatment of fungal infections in neonates in future.

**Methods:**

The clinical data of IFI neonates who received treatment in a tertiary NICU of China from January 2009 to December 2022 were retrospectively analyzed, including causative pathogens and the incidence of EOD. The neonates were divided into EOD group and non-EOD (NEOD) group. The general characteristics, risk factors and clinical outcomes of the two groups were compared.

**Results:**

Included in this study were 223 IFI neonates (137 male and 86 female) with a median gestational age (GA) of 30.71 (29,35) weeks and a median birth weight (BW) of 1470 (1120,2150) g. Of them, 79.4% were preterm infants and 50.2% were born at a GA of ≥ 28, <32 weeks, and 37.7% with BW of 1000–1499 g. Candida albicans (C. albicans) was the most common Candida spp. in these neonates, accounting for 41.3% of all cases, followed by C. parapsilosis (30.5%) and C. glabrata (7.2%). EOD occurred in 40 (17.9%) of the 223 cases. Fungal meningitis was the most common EOD, accounting for 13.5% of the 40 EOD cases. There was no significant difference in the premature birth rate, delivery mode, GA and BW between EOD and NEOD groups, but the proportion of male infants with EOD was higher than that without. There was no significant difference in antenatal corticosteroid use, endotracheal intubation, invasive procedures, use of antibiotics, total parenteral nutrition, blood transfusion, postnatal corticosteroid use, fungal prophylaxis and the incidence of necrotizing enterocolitis between the two groups, but the proportion of C. albicans infection cases in EOD group was higher than that in NEOD group (57.5% vs. 37.7%). Compared with NEOD group, the proportion of cured or improved infants in EOD group was significantly lower (*P* < 0.05), and the number of infants who died or withdrew from treatment was larger (*P* < 0.05).

**Conclusions:**

Our retrospective study showed that preterm infants were prone to fungal infection, especially very preterm infants. C. albicans was the most common Candida spp. for IFI, and was a high-risk factor for EOD. EOD can occur in both full-term and premature infants, so the possibility of EOD should be considered in all infants with IFI.

## Introduction

Invasive fungal infection (IFI) is an important form of neonatal infections, and one of the leading causes of morbidity and mortality in neonates, especially preterm and very low birth weight (VLBW) infants [1]. In China, the birth rate of preterm infants, especially extremely low birth weight (ELBW) infants, has been increasing gradually due to advanced maternal age and increased treatment for infertility [2] and the survival rate of ELBW infants has improved from 56.4% in 2010 to 68.0% in 2019 owing to the improvement in perinatal management of preterm neonates [3]. Consequently, fungal infection has become an increasing problem among infants in the neonatal intensive care unit (NICU).

The incidence of IFI varies with the practice setting and patient population, ranging from 0.5 to 20% [4, 5]. The incidence is the highest among low birth weight infants, ranging from 2.6 to 13.2% in VLBW infants, and from 6.6 to 20.6% in ELBW infants [6]. The commonest species accounting for neonatal candidemia are Candida albicans (C. albicans) and Candida parapsilosis (C. parapsilosis), with smaller numbers due to C. glabrata, C. tropicalis, C. krusei and C. lusitaniae [7, 8].

Neonates are particularly susceptible to fungal infection, and risk factors for IFI encompass three main domains: immunocompromised hosts, disruption of epithelial barriers, and level of colonization [9]. A number of potential predisposing factors for the development of IFI have been identified, including prematurity, VLBW/ELBW, use of corticosteroids, endotracheal tubes, central venous catheters (CVC), necrotizing enterocolitis (NEC), abdominal surgeries, prolonged or broad-spectrum antibiotics, histamine-2-receptor antagonists, and intravenous lipid emulsions [10–13]. In addition, birth weight (BW) and gestational age (GA) are the most important factors. Mortality attributable to IFI in neonates ranges from 25 to 55%, with neurodevelopmental impairment (NDI) reported up to 57% [14, 15]. Nearly 70% ELBW infants are reported to die from or experience severe NDI after IFI despite treatment [16]. End-organ damage (EOD) caused by neonatal IFI mainly refers to meningitis, endophthalmitis, brain parenchyma invasion, endocarditis, renal abscesses, positive cultures from other normally sterile body fluids, or hepatosplenic abscesses [17], which often lead to death or sequelae in infants. About 22% of infants with EOD to have cerebral palsy [18], and about 50% of infants with meningitis had NDI at 18 to 24 months corrected age [19]. Due to the difficulty of treatment, poor prognosis and medical burden, EOD infants often withdraw from treatment. However, there is a lack of the latest data on the incidence, causative pathogens, risk factors and clinical outcomes of IFI in EOD neonates in China. The aim of the present study is to summarize data on the epidemiology, risk factors, causative pathogens, and clinical outcomes of IFI-associated EOD among neonates in China by retrospective analysis of fungal infections in neonates in a tertiary center in China over a 14-year period for the sake of providing references for fungal prevention and treatment of neonates in future.

## Materials and methods

### Subjects of the study

This retrospective study protocol follows the principle stated in the Declaration of Helsinki, and the study was approved by the research ethics board of the Seventh Medical Center of the Chinese PLA General Hospital (Beijing, China), with a waiver of informed consent from this ethics board because of the retrospective design of the study. The subjects were consecutive IFI infants admitted in the tertiary neonate intensive care unit (NICU) of the said center from January 2009 to December 2022.

### Diagnostic criteria and definition

The diagnostic criteria of IFI follow the “Revised definitions of invasive fungal disease from the European Organization for Research and Treatment of Cancer/Invasive Fungal Infections Cooperative Group and the National Institute of Allergy and Infectious Diseases Mycoses Study Group (EORTC/MSG) Consensus Group” [20]. The definition of EOD in this article refers to Candida invasion of one or more organ systems, causing endophthalmitis, meningitis, brain parenchyma abscesses and ventriculitis, endocarditis, positive urine cultures, renal abscess and hepatosplenic abscess, which requires positive cerebrospinal fluid (CSF) culture, radiographic evidence, ophthalmologic examination, echocardiography findings, or echogenic findings, respectively [17]. After being diagnosed, IFI and EOD were treated with conventional antifungal therapy, and intravenous fluconazole, voriconazole, or amphotericin B was administered based on drug sensitivity test results.

### Inclusion and exclusion criteria

Fungal D-glucan and fungal culture were used in lab for candida detection. The inclusion criteria were infants diagnosed with proven IFI meeting the diagnostic criteria. The exclusion criteria were infants who were not diagnosed with IFI, or probable IFI, or possible IFI, and those with no complete case data.

### Data collection

According to their EOD status, the included neonates were classified into EOD group (*n* = 40) and non-EOD (NEOD) group (*n* = 183). Clinical data of the infants were retrospectively collected, including gender, GA, BW, perinatal conditions (premature rupture of membranes, antenatal corticosteroids, delivery modes, asphyxia, maternal diseases, et al.), major diseases (NEC, retinopathy of prematurity, bronchopulmonary dysplasia, intraventricular hemorrhage, patent ductus arteriosus, et al.), clinical interventions (endotracheal tubes, lumbar puncture, thoracentesis, Ommaya sac implantation, patent ductus arteriosus ligation, et al.), risk factors for IFI, causative pathogens, EOD and clinical outcomes.

### Statistical analysis

After data collection, SPSS 26.0 statistical software (IBM Corp., Armonk, NY, USA) was used for data analysis, and data analysis was performed from August 2023 to December 2023. Counting data including GA at birth and BW are expressed as median and quartile (Q1, Q3). Categorical data including Candida species distribution, risk factors and therapeutic outcomes are presented as frequencies and percentages, and differences between the EOD group and NEOD group were tested by chi-square test, with *P* < 0.05 as the difference having statistical significance, and all *P* values were 2-sided.

## Results

### Distribution of the Candida species

A total of 86,259 infants were admitted to our hospital from January 2009 to December 2022, of whom 223 were diagnosed with IFI, with an infection rate of 0.26%. In the 223 infants, C. albicans was the most common Candida spp., accounting for 41.3%, followed by C. parapsilosis (30.5%), C. glabrata (7.2%), C. saccharomyces (5.8%), C. famata (4.9%), and C. Portuguese (4%). Infants infected by other Candida spp. like C. guilliermondii, C. tropicalis and C. krusei were rare (1–3 cases for each species). Of the 223 IFI infants, 40 (17.9%) were diagnosed with EOD, including 23 (57.5%) infants infected by C. albicans, and 17 (42.5%) infected by other fungal species. Distribution of the C. species is shown in Table 1.

**Table 1:** Distribution of the Candida species

Items	No. (%)
C. albicans	92(41.3)
C. parapsilosis	68(30.5)
C. glabrata	16(7.2)
C. saccharomyces	13(5.8)
C. famata	11(4.9)
C. Portuguese	9(4)
C. guilliermondii	3(1.3)
C. tropicalis	3(1.3)
Trichosporon asahii	2(0.9)
Cryptococcus lorentis	2(0.9)
Pichia pastoris	1(0.4)
C. corneae	1(0.4)
C. krusei	1(0.4)
C. haemulonii	1(0.4)
Total	223(100)

### EOD and C. species distribution

Fungal meningitis was the most common EOD in these infants, accounting for 13.5% of all IFI cases, followed by fungal meningitis complicated with fungal endophthalmitis (1.8%), fungal urinary tract infection (1.3%), fungal endophthalmitis (0.9%), and fungal osteomyelitis (0.4%). C. albicans was the most common pathogen for EOD (*n* = 23), followed by C. glabrata (*n* = 6), C. parapsilosis (*n* = 5), C. famata(*n* = 2), C. saccharomyces (*n* = 1), C. Portuguese lusitaniae (*n* = 1), C. tropicalis (*n* = 1), and C. krusei (*n* = 1). EOD and C. species distributions are shown in Table 2.

**Table 2:** EOD and C. species distribution

	Fungal meningitis	Fungal meningitis complicated with fungal endophthalmitis	Fungal urinary tract infection	Fungal endophthalmitis	Fungal osteomyelitis	Total
C. albicans	16	4	3	0	0	23
C. glabrata	5	0	0	0	1	6
C. parapsilosis	5	0	0	0	0	5
C. famata	1	0	0	1	0	2
C. saccharomyces	1	0	0	0	0	1
C. Portuguese lusitaniae	1	0	0	0	0	1
C. tropicalis	1	0	0	0	0	1
C. krusei	0	0	0	1	0	1
Total	30	4	3	2	1	40

EOD, end-organ damage

### The trend of EOD and IFI in recent 14 years

**Fig. 1:**
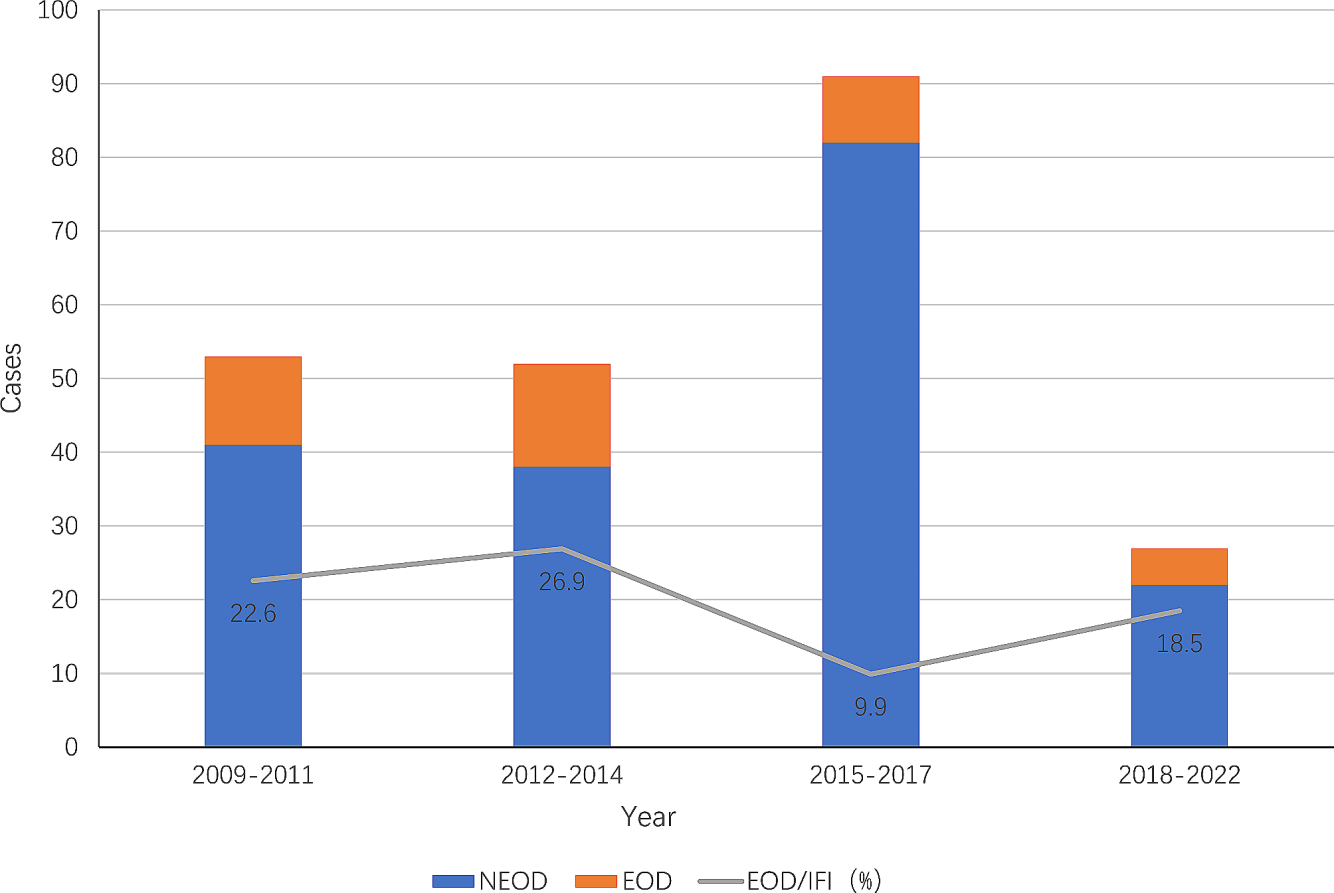
The trend of EOD and IFI in recent 14 years

We divide the 14 years into four time periods: 2009–2011, 2012–2014, 2015–2017 and 2018–2022, with 20,385, 21,802, 21,370 and 22,702 infants hospitalized respectively. Of the infants hospitalized, 53, 52, 91, 27 infants were diagnosed with IFI and 12, 14, 9, 5 infants were diagnosed with EOD in the four periods respectively. The infection rate of IFI were 0.26%, 0.24%, 0.43%, 0.12% and the proportions of EOD/IFI were 22.6%, 26.9%, 9.9%, 18.5% in the four periods respectively. The trend of EOD and IFI in recent 14 years are shown in Fig. 1.

### Characteristics of EOD and NEOD infants

Of the 223 included infants, 137 were male and 86 were female with a median GA of 30.71 (29,35) weeks and a median BW of 1470 (1120,2150) g. Of them, 13.9% were born with a GA of < 28 weeks, 50.2% with a GA of ≥ 28, <32 weeks, 14.8% with a GA of ≥ 32, <37 weeks, 21.1% with a GA of ≥ 37 weeks. 14.3% were born with BW of < 1000 g, 37.7% with BW of 1000–1499 g, 28.3% with BW of 1500–2499 g, 19.7% with BW of ≥ 2500 g. Most of them (177, 79.4%) were preterm infants. Of the 223 infants, 109 (48.9%) were born by cesarean section and the rest were born by vaginal delivery. The differences in gender, premature birth, delivery mode, GA and BW between the EOD and NEOD groups were tested by Chi-square test. There was no significant difference in premature birth, delivery mode, GA and BW between the two groups, but the proportion of male infants in EOD group was higher than that in NEOD group (*p* = 0.03). Comparisons of the characteristics of the two groups are shown in Table 3.

**Table 3:** Characteristics of EOD and NEOD infants

Characteristics	EOD, *n* (%) (*n* = 40)	NEOD n (%), (*n* = 183)	*P* Value
Gender			
Male	33(82.5)	104(56.8)	0.003
Female	7(17.5)	79(43.2)	
Mature/Premature			
Premature	30(75.0)	147(80.3)	0.451
Mature	10(25.0)	36(19.7)	
Delivery mode			
Vaginal delivery	22(55.0)	92(50.3)	0.588
cesarean section	18(45.0)	91(49.7)	
Gestational age			
< 28	7(17.5)	24(13.1)	0.208
≥ 28, < 32	14(35.0)	98(53.6)	
≥ 32, < 37	8(20.0)	25(13.7)	
≥37	11(27.5)	36(19.7)	
Birth Weight			
< 1000 g	5(12.5)	27(14.8)	0.753
1000–1499 g	13(32.5)	71(38.8))	
1500–2499 g	12(30.0)	51(27.9)	
≥ 2500 g	10(25.0)	34(18.6)	

EOD, end-organ damage; NEOD, non end-organ damage

### Risk factors contributing to EOD

Antenatal corticosteroids were used in 93 (41.7%) of the 223 IFI infants, and endotracheal intubation was performed in 204 (91.5%) of them, and central venous catheterization (CVC) was performed in 168 (75.35%) of them. Of them, 213 (95.5%) underwent invasive procedures such as lumbar puncture, thoracentesis, Ommaya sac implantation, patent ductus arteriosus ligation, retinopathy of prematurity treatment, and abdominal surgery. Antibiotics were administered to 195 (87.4%) of the infants. The percentage of IFI infants using total parenteral nutrition (TPN) and blood transfusion were 89.2% and 84.3%, respectively. Postnatal corticosteroids were used in 22.4% of the infants, and fungal prophylaxis was administered to 52.9% of the infants. 10.8% of the infants suffered from NEC.

Risk factor distribution between EOD and NEOD groups was compared by Chi-square test. The results showed that the proportion of C. albicans infection cases in EOD group was significantly higher than that in NEOD group (*P* = 0.021). There was a significant difference in the percentage of IFI infants receiving CVC between the two groups (*P* = 0.013), while there was no significant difference in the use of antenatal corticosteroids, endotracheal intubation, invasive procedures, use of antibiotics, TPN, blood transfusion, postnatal corticosteroids, fungal prophylaxis, and the incidence of NEC between the two groups. The details are shown in Table 4.

**Table 4:** Risk factors contributing to EOD

Characteristics	EOD, n (%) (*n* = 40)	NEOD, n (%), (*n* = 183)	*P* Value
Antenatal corticosteroids			
Yes	15(37.5)	78(42.6)	0.552
No	25(62.5)	105(57.4)	
Endotracheal tubes			
Yes	37(92.5)	167(91.3)	0.799
No	3(7.5)	16(8.7)	
CVC			
Yes	4(10)	53(29)	0.013
No	36(90)	130(71)	
Invasive procedures			
Yes	40(100)	173(94.5)	0.130
No	0(0)	10(5.5)	
Antibiotics			
Yes	35(87.5)	160(87.4)	0.991
No	5(12.5)	23(12.6)	
TPN			
Yes	36(90)	163(89.1)	0.864
No	4(10)	20(10.9)	
Blood transfusion			
Yes	36(90)	152(83.1)	0.274
No	4(10)	31(16.9)	
Postnatal corticosteroids			
Yes	10(25)	40(21.9)	0.666
No	30(75)	143(78.1)	
Fungal prophylaxis			
Yes	23(57.5)	95(51.9)	0.521
No	17(42.5)	88(48.1)	
NEC			
Yes	5(12.5)	19(10.4)	0.695
No	35(87.5)	164(89.6)	
C. albicans			
Yes	23(57.5)	69(37.7)	0.021
No	17(42.5)	114(62.3)	

EOD, end-organ damage; NEOD, non end-organ damage; CVC, central venous catheters; TPN, total parenteral nutrition; NEC, necrotizing enterocolitis

### Therapeutic outcomes of EOD and NEOD infants

The therapeutic outcomes were classified as cured, improved, death and withdrawal. Cured: the symptoms and signs disappeared and the laboratory test results were normal. Improved: the symptoms and signs were improved but oral medication must be continued after discharge. Death: the infant died during hospitalization. Withdrawal: termination of treatment at the parent’s request. Most IFI infants (175/223, 78.5%) were cued. Thirty-three infants (14.8%) withdrew from treatment due to the concern or apprehension of their parents about the poor prognosis. Fourteen infants (6.3%) were discharged when their conditions improved, and oral medication was continued after discharge until they were cured. One infant died in the hospital. The differences in therapeutic outcomes between the two groups was checked by chi-square test. The proportion of cured infants was lower, and the proportion of infants who died or withdrew from treatment in EOD group was significantly higher than that in NEOD group (*P* < 0.01). The details are shown in Table 5.

**Table 5:** Therapeutic Outcomes of EOD and NEOD infants

Therapeutic Outcomes	EOD, *n* (%) (*n* = 40)	NEOD, *n* (%), (*n* = 183)	*P* Value
Cured	18(45)	157(85.8)	< 0.01
improved	4(10)	10(5.4)	
Death	1(2.5)	0	
Withdrawal	17(42.5)	16(8.7)	

EOD, end-organ damage; NEOD, non end-organ damage

## Discussion

As a major problem in NICU infants, IFI may cause EOD, which increases mortality and disability of the infants. Although research on EOD caused by IFI is very important, there are few relevant researches. In this article, we retrospectively analyzed the characteristics, risk factors, causative pathogens, EOD and therapeutic outcomes of 223 IFI infants over a decade, and found that most of them were premature infants, more than a half were very preterm infants, and most infants had risk factors for IFI. C. albicans was the most common Candida spp. Fungal meningitis was the most common EOD. Most infants achieved good therapeutic outcomes. However, EOD infants were more likely to withdraw from treatment due to the poor prognosis that some parents were concerned about.

Candida species that colonize the gut and the skin cause either localized, deep-seated infection, or candidemia. Candidemia leads to colonization and biofilm formation. Once candidemia has developed, the fungal species may disseminate, leading to deep-tissue infections in the heart, brain, eye, liver, lung, spleen, kidney and/or bone, causing EOD. When the fungus in the bloodstream enters urine, candiduria may occur [21]. It was found in the present study that C. albicans is the main pathogenic fungus, accounting for 41.3% of all IFI cases in our series, especially in EOD group, accounting for 57%. The proportion of C. albicans infection cases in EOD group was significantly higher than that in NEOD group, suggesting that C. albicans is more likely to cause EOD. C. albicans can invade the central nervous system. Although the blood brain barrier is made of specialized brain microvascular endothelial cells which are efficient in limiting the transcellular flux, C. albicans cells can cross brain microvascular endothelial cells in vitro, without affecting the monolayer integrity [22]. From previous studies, we infer that the neonatal brain is susceptible to fungal infection. The present study also showed that meningitis was the most common complication of fungal infection.

Potential factors for IFI have been identified. BW and GA are important risk factors [15, 23]. Other major risk factors include exposure to broad-spectrum antibiotics, the duration of parenteral nutrition, use of fat emulsions, prolonged artificial ventilation, immunodepression, fungal colonization, application of CVC and ICU admission [12, 24–26]. In our study, the percentage of IFI infants with endotracheal intubation, CVC, invasive procedures and the use of broad-spectrum antibiotics was high, which is consistent with the results of above studies. In this study, we tried to see whether these risk factors for IFI, such as BW and GA, were also risk factors for EOD, but the results were not as expected. Hanin et al. found that persistent Candida growth in blood cultures, prematurity and long-term antibiotic use are significant risk factors for EOD, which prolongs the length of hospital stay and increases the neonatal morbidity [18]. However, in the present study, no significant correlation was found between prematurity and EOD, suggesting that both premature or full-term infants with IFI should be alert to the possibility of EOD. We also found that the proportion of male infants in EOD group was higher than that in NEOD group, which may also be suspected as the risk for EOD. But as no previous study has reported this association, further study is required to verify the correlation between gender and EOD.

CVC is known to be associated with bloodstream infection, a devastating infection associated with high morbidity and mortality [27, 28]. We found that CVC was used in 75.3% of the IFI infants in our series. This high percentage of CVC use was probably associated with fungal infection as reported in previous studies. Carter et al. reported a case of a neonate born at 26^+ 4^ weeks of GA due to respiratory immaturity. The neonate developed fever and lethargy during hospitalization, and CSF and peripheral blood cultures yielded C. parapsilosis, confirming that the infection was related to CVC, which calls for awareness of complications resulting from CVC [29]. A retrospective cohort analysis by Fisher et al. showed that 30 (10%) of their 285 patients died within 30 days after admission, and CVC retention was associated with a significant increased risk of death (OR, 2.50; 95% CI, 1.06–5.91). Their data suggest that early CVC removal should be considered [30]. Previous studies have shown that CVC is significantly associated with fungal infection, but no study has reported the correlation between CVC and EOD. The present study showed that the proportion of patients who used CVC in EOD group was smaller than that in NEOD group. Based on this finding, we tend to conclude that CVC is a risk factor for IFI, but not for EOD. Further studies with larger samples are needed to confirm this finding.

Moreover, we found that there were no significant differences in BW, antenatal corticosteroid use, endotracheal intubation, invasive procedures, use of antibiotics, TPN, blood transfusion, postnatal corticosteroid use, fungal prophylaxis and NEC as the risk factors between EOD and NEOD groups, suggesting that these factors not necessarily risk factors for EOD. However, C. albicans was significantly associated with EOD, suggesting that EOD is related to special fungal species. For C. albicans infection, more and epical attention should be paid to the possibility of EOD, whether premature or full-term infants.

In recent years, some studies have reported the prognosis of neonatal fungal infection. A multinational retrospective study in Europe showed that all-cause mortality of IFI pediatric patients was 14.4%, and the mortality of neonates, infants and older children was 18.2%, 14.5% and 11.5%, respectively. Compared with older children, no significant difference was found between neonates and infants [31]. Our study found an overall mortality rate of 15.2% (including one death and 33 who withdrew from treatment), which are similar to the European study. The European study also showed that the mortality rates were similar for C. albicans (13.6%) and C. parapsilosis (12.7%), while the mortality rates were higher for C. tropicalis (21.3%) and C. krusei (19.3%). We did not do a similar study because the number of deaths caused by IFI other than C. albicans and C. parapsilosis were small. A prospective multicenter study conducted in Spain found that VLBW infants showed a significantly high incidence of systemic candidiasis (4.8%) and C. albicans was the most frequent species (52.5%). The Spanish study also found that the mortality rate was 10.2% and all deaths occurred in the VLBW cohort with candidemia [32]. The lower mortality rate indicates that, in comparison to the treatment provided in developed countries, our level of treatment still requires significant enhancement. A retrospective study Saudi Arabia showed that a total of 14 (11%) out of 130 neonates with IFI had EOD, and mortality rate was 35.7% in EOD group and 28.4% in NEOD group with IFI [18]. This is consistent with our finding, which showed that the mortality rate in EOD group was significantly higher than that in NEOD group.

### Limitations

This study has some limitations. First, it is a retrospective study, and selection bias may be unavoidable. Additionally, the single center design may limit generalizability.

## Conclusions

In this retrospective study, we analyzed the clinical data and found that preterm infants were prone to fungal infection, especially very preterm infants. C. albicans was the most common Candida spp., and the proportion of C. albicans infection cases in EOD group was higher than that in NEOD group. Fungal meningitis was the most common EOD, and the mortality rate in EOD group was higher. It is crucial for neonatologists to promote the prevention, early detection and treatment of IFI in high-risk neonates, and pay particular attention to the possibility of EOD in all IFI infants.

## Data Availability

The datasets used and/or analysed during the current study available from the corresponding author on reasonable request.
